# VinDr-Mammo: A large-scale benchmark dataset for computer-aided diagnosis in full-field digital mammography

**DOI:** 10.1038/s41597-023-02100-7

**Published:** 2023-05-12

**Authors:** Hieu T. Nguyen, Ha Q. Nguyen, Hieu H. Pham, Khanh Lam, Linh T. Le, Minh Dao, Van Vu

**Affiliations:** 1 Institute of Big Data, Hanoi, Vietnam; 2 College of Engineering and Computer Science (CECS), VinUniversity, Hanoi, Vietnam; 3 VinUni-Illinois Smart Health Center, Hanoi, Vietnam; 4 Hospital 108, Department of Radiology, Hanoi, Vietnam; 5 Hanoi Medical University Hospital, Department of Radiology, Hanoi, Vietnam; 6 Yale University, Department of Mathematics, New Heaven, CT 06511 USA

**Keywords:** Image processing, Data publication and archiving

## Abstract

Mammography, or breast X-ray imaging, is the most widely used imaging modality to detect cancer and other breast diseases. Recent studies have shown that deep learning-based computer-assisted detection and diagnosis (CADe/x) tools have been developed to support physicians and improve the accuracy of interpreting mammography. A number of large-scale mammography datasets from different populations with various associated annotations and clinical data have been introduced to study the potential of learning-based methods in the field of breast radiology. With the aim to develop more robust and more interpretable support systems in breast imaging, we introduce VinDr-Mammo, a Vietnamese dataset of digital mammography with breast-level assessment and extensive lesion-level annotations, enhancing the diversity of the publicly available mammography data. The dataset consists of 5,000 mammography exams, each of which has four standard views and is double read with disagreement (if any) being resolved by arbitration. The purpose of this dataset is to assess Breast Imaging Reporting and Data System (BI-RADS) and breast density at the individual breast level. In addition, the dataset also provides the category, location, and BI-RADS assessment of non-benign findings. We make VinDr-Mammo publicly available as a new imaging resource to promote advances in developing CADe/x tools for mammography interpretation.

## Background & Summary

Breast cancer is among the most prevalent cancers and accounts for the largest portion of cancer deaths, with an estimated 2.2 million new cases in 2020^1^. Treatment is most successful when breast cancer is at its early stage. Biennial screening can reduce breast cancer mortality rate by 30%^2^. Among standard imaging examinations for breast cancer diagnosis, namely mammography, ultrasound, digital breast tomosynthesis, and magnetic resonance, mammography is the recommended modality for cancer screening^3^. Interpreting mammography for breast cancer screening is a challenging task. The recall rate of mammogram screening is around 11% with a sensitivity of 86.9%, while the cancer detection rate is 5.1 per 1,000 screens^4^. It means that a large portion of cases called back for further examinations eventually result in non-cancer. Improving cancer screening results may help reduce the cost of follow-up examinations and unnecessary mental burdens on patients.

With recent advancements of learning-based algorithms for image analysis^5,6^, several works have adapted deep learning networks for mammography interpretation and showed potential to use in clinical practices^7–12^. In retrospective settings, the CAD tool as an independent reader can achieve a performance comparable to an average mammographer^8^. It can be leveraged as a decision support tool that helps enhance radiologists’ cancer detection with the reading time being unchanged^9^. In another human-machine hybrid setting, where radiologists and machine-learning algorithm independently estimate the malignancy of the lesions, the linear combination of human and machine prediction show higher performance than a single human or machine reader^10^. The improvement as a result human-machine combination is also witnessed in screening mammography interpretation^11^. Furthermore, there was evidence that shows a machine learning model developed by training on data from a specific population (UK) can generalize and perform well on another population (US)^12^.

The recent progress in the study of mammography interpretation has drawn much attention with an increasing number of mammogram datasets with various characteristics, while some datasets are publicly available to the research community, some have restricted access or are not open^13–19^ (see Table 1). Digital Database for Screening Mammography (DDSM)^18^ and Mammographic Image Analysis Society (MIAS) dataset^19^ are the two earliest public datasets that provide digitalized scans of screen-film mammograms with precise annotations of breast abnormalities. The MIAS dataset was released in 1994 with 161 studies collected in the United Kingdom while the DDSM dataset consisted of 2,620 exams collected from institutions in the United States. Compared to the former one, the DDSM dataset has a significantly larger scale and follows the BI-RADS standard. To the best of our knowledge, INbreast^17^, released in 2012 with 115 exams from Portugal, is the very first public dataset that provides digital mammograms with lesions annotations and overall exam assessment following the BI-RADS standard. In 2019, the NYU Breast Cancer Screening Dataset^16^ was introduced with 229,426 screening exams, consisting of 1,001,093 images, from 141,473 women screened at NYU Langone Health. The dataset contains breast-level cancer based on biopsy results, exam-level assessment of BI-RADS, breast density, and biopsied finding annotations. While the dataset is not public, the subsequent work^10^ based on this dataset showed evidence that a large-scale dataset of mammography can enable a computer-aided system that helps improve radiologist performance. At around the same time, the Cohort of Screen-Aged Women Case-Control (CSAW-CC)^20^ was opened for evaluating AI tools for breast cancer, including 1303 cancer cases and 10,000 randomly selected controls from Karolinska University Hospital. The CSAW-CC dataset is a subset of the full CSAW dataset including women screened in the Stockholm region between 2008 and 2015. In cancer cases, visible tumors in mammography were manually annotated on a pixel level. Another large-scale dataset is the OPTIMAM mammography image database^13^ (OMI-DB) which consists of images and clinical data of 172 282 women screened and diagnosed in several institutions in the United Kingdom since 2011. To access to the OMI-DB dataset, the research group must submit an application to elaborate the scientific purpose based on the dataset which will be reviewed by the OPTINAM steering committee. In addition, the Chinese Mammography Database was recently introduced, containing 1,775 studies from several Chinese institutions. All cases have breast-level benign and malignant confirmed by biopsy, and molecular subtypes are available for 749 cases. A summary of the characteristics of these datasets is given in Table 2.

**Table 1:** Summary of mammography datasets.

Dataset	Origin	Introduction year	#studies	#images	Finding type	Annotations	BI-RADS assessment	Breast density	Mode of acquisition
**MIAS**^19^	United Kingdom	1994	161	322	Mass, calcification, asymmetry, and distortion	Circle around the finding, specified by center and radius	No	Yes	SFM
**DDSM**^18^	United States	1999	2,620	10,480	Mass and Calcification	Contour enclosing the finding	Yes	Yes	SFM
**INBreast**^17^	Portugal	2012	115	410	Mass, calcification, asymmetry, and distortion	Contour enclosing the finding	Yes	Yes	FFDM
**NYU Dataset**^16^	United State	2019^†^	229,426	1,001,093	Biopsied lesions	Contour enclosing the finding	Yes	Yes	FFDM
**CSAW-CC**^14^	Sweden	2020	24,694	98,788	Visible tumors & tumor signs	Contour enclosing the finding	No	No	FFDM
**OMI-DB**^13^	United Kingdom	2021	NA	3,072,878^*^	Lesions	Rectangular region of interest	No	No	FFDM
**CMMD**^15^	China	2021	1,775	5,202	Biopsied abnomalities(mass or calcification	No local annotations	No	No	FFDM
**VinDr-Mammo**	Vietnam	2022	5,000	20,000	Mass, calcification, asymmetry, distortion, and other associated features	Rectangular region of interest	Yes	Yes	FFDM

^†^Not publicly accessible

*Including for-presentation and for-processing images

**Table 2:** Characteristics of the participating radiologists.

Annotator	Years’ experience	Annual Diagnostic Volume (studies)
Radiologist 1	14	10,000
Radiologist 2	21	15,000
Radiologist 3	22	15,000
Average	19	13,333

Mean annual diagnostic volumes were estimated based on the number of mammogram scans interpreting.

Along with the existing mammography datasets, we introduce and release the VinDr-Mammo dataset, an open-access large-scale Vietnamese dataset of full-field digital mammography consisting of 5,000 four-view exams with breast-level assessment and extensive lesion-level annotations. Our aims is to enhance the diversity of the publicly available mammography data for a more robust AI system and to lean towards a more interpretable system via extensive lesion-level annotations. Mammographies were acquired retrospectively from two primary hospitals in Hanoi, Vietnam, namely Hospital 108 (H108) and Hanoi Medical University Hospital (HMUH). Breast cancer assessment and density are reported following Breast Imaging Reporting and Data System^21^. Breast abnormalities that need short-term follow-up or are suspicious of malignancy are marked by bounding rectangles. Following European guideline^22^, mammography exams were independently double-read. Any discordance between the two radiologists would be resolved by arbitration with the involvement of a third radiologist. To the best of our knowledge, VinDr-Mammo is currently the largest public dataset (20,000 scans) of full-field digital mammography that provides breast-level BI-RADS assessment category along with suspicious or probably benign findings that need follow-up examination. By introducing the dataset, we contribute a benchmarking imaging dataset to evaluate and compare algorithmic support systems for breast cancer screening based on FFDM.

## Methods

**Fig. 1:**
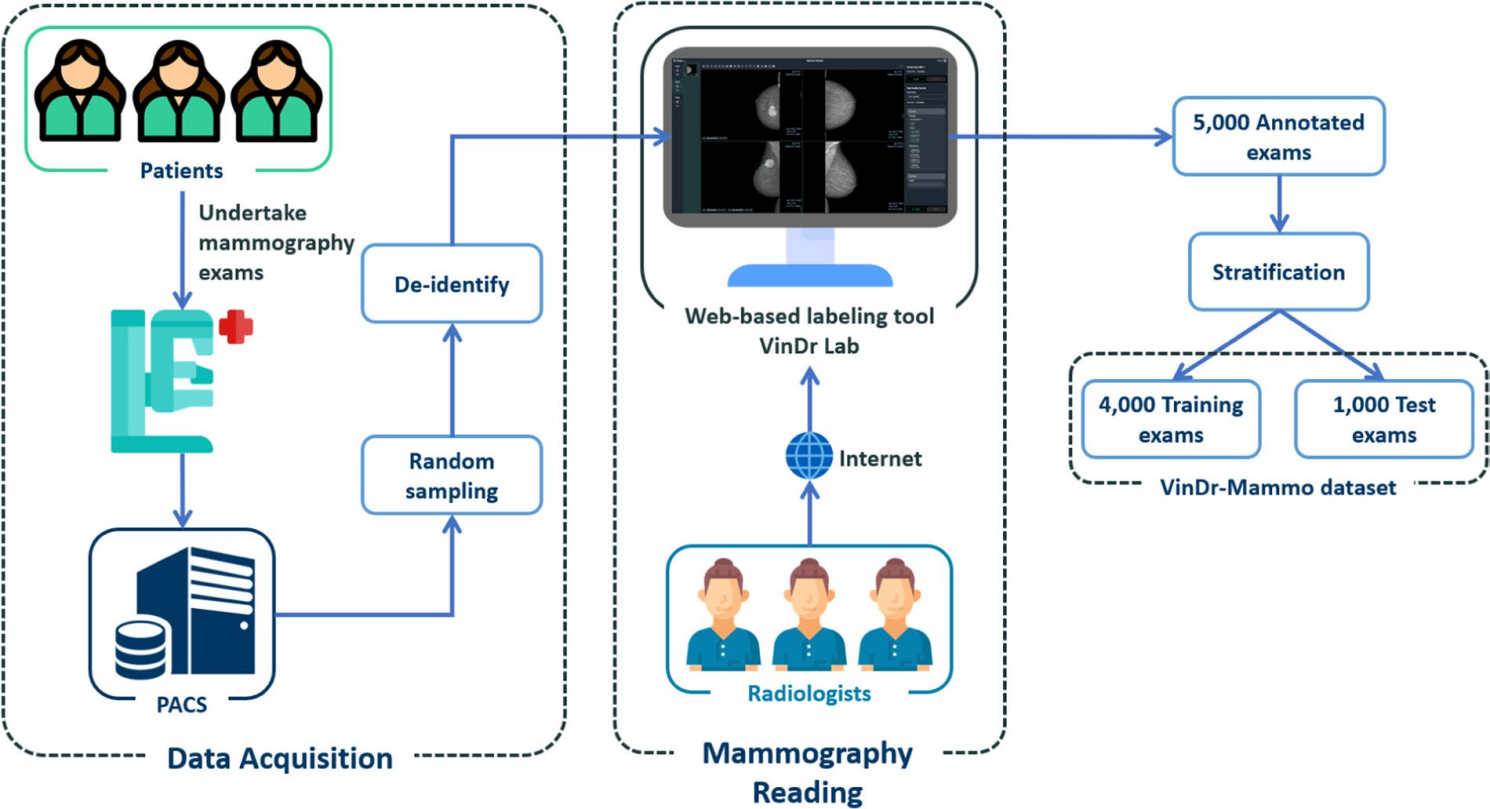
Overview of the data creation process. First, for-presentation mammograms in DICOM format were collected retrospectively from the hospital’s PACS. These scans then got pseudonymized to protect patient’s privacy. Next, the dataset was annotated by radiologists via a web-based labeling tool called VinDr Lab, which was developed to manage medical image labeling projects with dedicated features for the medical domain. Finally, the annotated exams were split into a training set of 4,000 exams and a test set of 1,000 exams.

This study was approved by the Institutional Review Board of the HMUH and H108. All the personally identifiable information and protected health information of patients were removed. Additionally, this project did not affect clinical care at these two hospitals; hence patient consent was waived. The creation of the VinDr-Mammo dataset involves three stages: data acquisition, mammography reading, and data stratification. An overview of the data creation process is illustrated in Fig. 1.

### Data acquisition

In this step, 20,000 mammography images in DICOM format from 5,000 mammography examinations were randomly sampled from the pool of all mammography examinations taken between 2018 and 2020 via the Picture Archiving and Communication System (PACS) of Hanoi Medical University Hospital (HMUH–https://hmu.edu.vn/) and Hospital 108 (H108–https://www.benhvien108.vn/home.htm). As the exams were randomly selected, the dataset includes both screening and diagnostic exams and represents the real distribution of patient cohorts in these hospitals. All images have the image presentation intent type of “FOR PRESENTATION” as those of for-processing were not stored by the hospitals. Images were acquired on equipments from 3 vendors, namely SIEMENS, IMS, and Planmed. All radiographers working at these hospitals were trained and certified by HMUH. To ensure patient privacy is protected, identifiable patient information in DICOM tags is fully removed via a Python script. Only necessary information used for loading and processing DICOM images and patient demographic information, i.e., age, is retained. Besides DICOM meta-data, associated information might appear in the images, such as laterality and view of the image and sometimes the patient’s name. As this textual information usually appears in the corners of the image, we remove them by setting to black all pixels in a rectangle at each corner. The size of the rectangle is determined by visually inspecting a subset of the collected dataset. To validate the pseudonymization stage, both DICOM metadata and image are manually reviewed by human readers.

### Mammography reading

This dataset aims to provide both the overall assessment of the breast and information of local-level findings, which are essential to developing CADx and CADe systems for breast cancer screening. To this end, the 5,000 sampled exams containing 20,000 images were re-read, as the associated radiology reports do not indicate the exact locations of the findings.

**Fig. 2:**
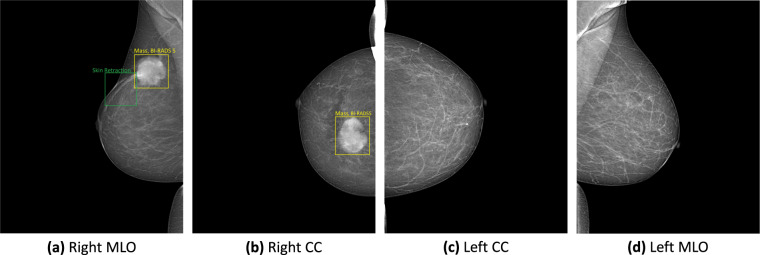
A sample mammography exam with the right breast assessed with BI-RADS 5, density B and the left breast with BI-RADS 1, density B. CC denotes craniocaudal and MLO denotes mediolateral oblique.

The reading results follow the schema and lexicon of the Breast Imaging Reporting and Data System^21^. At the breast level, the overall BI-RADS assessment categories and breast density level (also termed breast composition) are provided. There are seven BI-RADS assessment categories, namely BI-RADS 0 (need additional imaging or prior examinations), BI-RADS 1 (negative), BI-RADS 2 (benign), BI-RADS 3 (probably benign), BI-RADS 4 (Suspicious), BI-RADS 5 (highly suggestive of malignancy) and BI-RADS 6 (known biopsy-proven). Since the biopsy results are not available, there is no presence of BI-RADS 6 in the re-reading process. Regarding the breast density level, its four categories are A (almost entirely fatty), B (scattered areas of fibroglandular), C (heterogeneously dense), and D (extremely dense). For the mammography findings, the list of findings provided in this dataset includes the mass, calcification, asymmetries, architectural distortion, and other associated features, namely suspicious lymph node, skin thickening, skin retraction, and nipple retraction. Each finding is marked by a bounding box to localize the abnormal finding. In the given finding list, BI-RADS assessment is provided for mass, calcification, asymmetries, and architectural distortion. Since the purpose of this dataset is for breast cancer screening, benign findings, i.e., findings of BI-RADS 2, are not reported to reduce the annotating time. Only findings of BI-RADS categories greater than 2, which are not confident of benign or likely to be malignant, are marked. More details of the reading reports are provided in supplementary materials. Figure 2 illustrates a sample mammography exam with both finding annotations and breast-level assessments reported by radiologists.

The mammography reading process was facilitated by a web-based annotation tool called VinDr Lab (https://github.com/vinbigdata-medical/vindr-lab), which was specifically designed for viewing and annotating medical images with the medical image viewer being based on the Open Health Imaging Foundation project (https://ohif.org/). The three participating radiologists were able to remotely access the data for reading and annotating. All three radiologists hold healthcare professional certificates, which require up to eight years of training program, and are approved by the Joint Review Committee on Education in Radiology, Vietnamese Ministry of Health. Furthermore, each reader has more than ten years of experience in the field. Specifically, all three radiologists received training in mammography interpretation and had an average of 19 years of clinical experience interpreting mammography (range 14–22 years). Additionally, each annotator reviewed an average of 13,333 mammography exams annually (range 10,000–15,000). Table 2 shows the characteristics of the radiologists who participated in our data annotation process.

Each mammography exam was then assigned to two radiologists and read independently. In cases of discordance, the exam would be assigned to the third radiologist at a higher senior experience level to make the final decision taking into account annotations of previous readers. After completing the reading process, the breast-level categories and local annotations were exported in JavaScipt Object Notation (JSON) format. Subsequently, we parsed the exported file to discard unnecessary information, namely annotation timestamp, and radiologist’s identifier then simplified the file’s structure and transformed it to comma-separated values (CSV) file so that it could be easily parsed.

### Data stratification

**Fig. 3:**
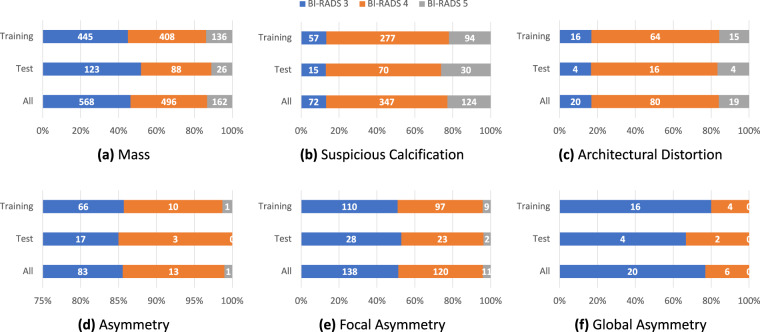
Statistics of BI-RADS assessment of findings.

**Fig. 4:**
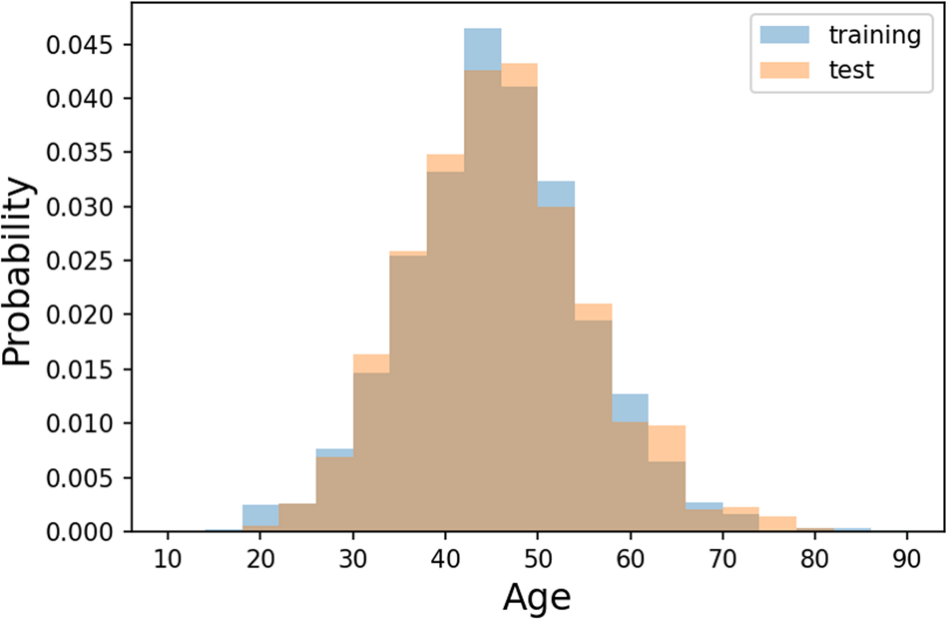
Distribution of patient age. This statistic is calculated overall all exams in which patient’s age is available.

Recent CADx and CADe solutions are mostly learning-based approaches that require separating the dataset into disjoint subsets for training and evaluation. A pre-defined training/test split would help guarantee that different research works will use the same exams for training and testing. Otherwise, inconsistent or unstated splits in different research works might hinder the reproducibility and comparison of these works. For an appropriate stratification, both the training and test sets should reflex the assessment, composition, and distribution of findings of the whole dataset. However, stratifying that dataset while preserving the correlation between various data characteristics is challenging as the number of combinations of different attributes grows exponentially with the number of attributes (in this case BI-RADS, breast composition, and findings categories). Hence, we split the dataset by an algorithm called iterative stratification^23^ which bases on a relaxed target that only retains a fraction of the appearance of each attribute while ignoring their co-occurrence. One-fifth of the dataset, equivalent to 1,000 exams, is for testing and the rest for training. The attributes that are taken into account for splitting include breast-level BI-RADS categories, breast composition, findings categories, and the attached BI-RADS categories (if any). The distribution of breast-level BI-RADS categories, breast composition, and findings for each subset are provided in Tables 3–5, respectively. The BI-RADS assessment of finding and patient age distribution are also depicted in Figs. 3, 4.

**Table 3:** Statistics of breast-level BI-RADS assessment.

	Breast BI-RADS					Total
	1	2	3	4	5	
**Training**	5,362 (67.03%)	1,871 (23.39%)	372 (04.65%)	305 (03.81%)	90 (01.12%)	8,000
**Test**	1,341 (67.05%)	467 (23.35%)	93 (04.65%)	76 (03.80%)	23 (01.15%)	2,000
**Overall**	6,703 (67.03%)	2,338 (23.38%)	465 (04.65%)	381 (03.81%)	113 (01.13%)	10,000

**Table 4:** Statistics of breast density.

	Breast Density				Total
	A	B	C	D	
**Training**	40 (00.50%)	764 (09.55%)	6,116 (76.45%)	1,080 (13.50%)	8,000
**Test**	10 (00.50%)	190 (09.50)	1,530 (76.50%)	270 (13.50%)	2,000
**Overall**	50 (00.50%)	954 (09.54%)	7,646 (76.46%)	1,350 (13.50%)	10,000

**Table 5:** Findings statistics on the VinDr-Mammo dataset.

Finding	Split		Total
	Training	Test	
Mass	989 (6.181)	237 (5.925)	1,226 (6.130)
Suspicious Calcification	428 (2.675)	115 (2.875)	543 (2.715)
Asymmetry	77 (0.481)	20 (0.500)	97 (0.485)
Focal Asymmetry	216 (1.350)	53 (1.325)	269 (1.345)
Global Asymmetry	20 (0.125)	6 (0.150)	26 (0.130)
Architectural Distortion	95 (0.594)	24 (0.600)	119 (0.595)
Skin Thickening	45 (0.281)	12 (0.300)	57 (0.285)
Skin Retraction	15 (0.094)	3 (0.075)	18 (0.090)
Nipple Retraction	30 (0.188)	7 (0.175)	37 (0.185)
Suspicious Lymph Node	46 (0.288)	11 (0.275)	57 (0.285)

The number of findings and the rate of findings per 100 images are provided for the training set, test set, and the whole dataset.

## Data Records

Both DICOM images and radiologists’ annotations of the dataset are available on PhysioNet^24^ for public access. Breast-level and lesion-level annotations of the whole dataset are stored in CSV files breast-level_annotations.csv and finding_annotations.csv, respectively. The images are structured into subfolders according to the encoded study identifiers, each of which contains four images corresponding to four views of the exam. The subfolder name and image file name are named following the study identifier and image identifier. The information of the breast-level annotations is provided for each image even though there is redundancy since each breast is associated with two images of different view positions, i.e., MLO and CC. We find this representation more convenient because other metadata of the image, namely laterality and view position, can also be included, eliminating the need to parse this information from the DICOM tags. Metadata for each image in the breast-level_annotations.csv file includes:

- study_id: The encoded study identifier.
- series_id: The encoded series identifier.
- image_id: The encoded image identifier.
- laterality: Laterality of the breast depicted in the image. Either L or R.
- view_position: Breast projection. Standard views are CC and MLO.
- height: Height of the image.
- width: Width of the image.
- breast_birads: BI-RADS assessment of the breast that the image depicts.
- breast_density: Density category of the breast that the image depicts.
- split: Indicating the split to which the image belongs. Either training or test.

Regarding breast findings, each annotation represents the occurrence of breast abnormality at a region, represented by a bounding box, in a specific image. This means that a single finding may associate with annotations from different views, yet this linking information is not acquired in the annotation process. Metadata for each finding annotation in the finding_annotations.csv file contains:

- image_id: The encoded identifier of the image in which the finding appears.
- study_id: The encoded identifier of the associated study.
- series_id: The encoded identifier of the associated series.
- laterality: Laterality of the breast in which the finding appears.
- view_position: Orientation with respect to the breast of the image.
- height: Height of the image.
- width: Width of the image.
- breast_birads: BI-RADS assessment of the breast that the image depicts.
- breast_density: Density category of the breast that the image depicts.
- finding_categories: List of finding categories attached to the region, e.g., mass with skin retraction.
- finding_birads: BI-RADS assessment of the marked finding.
- xmin: Left boundary of the box.
- ymin: Top boundary of the box.
- xmax: Right boundary of the box.
- ymax: Bottom boundary of the box.
- split: Indicating the split to which the image belongs. Either training or test.

## Technical Validation

The data pseudonymization procedure and the quality of the labeling process were strictly controlled. First, all meta-data was manually reviewed to ensure that all individually identifiable health information or PHI^25^ of the patients has been fully removed to meet data privacy regulations such as the U.S. HIPAA^26^ and the European GDPR^27^. In addition, the image content of all mammograms was manually reviewed case-by-case by human readers to ensure that no patient information remained. We developed a set of rules underlying our labeling tool to reduce mislabeling. These rules allowed us to verify the radiologist-generated labels automatically. Specifically, they prevent annotators from mechanical mistakes like forgetting to choose global labels or marking lesions on the image while choosing “BI-RADS 1” as the breast-level assessment.

## Usage Notes

The VinDr-Mammo dataset was created for the purpose of developing and evaluating computer-aided detection and diagnosis algorithms based on full-field digital mammography. In addition, it can also be used for general tasks in computer vision, such as object detection and multiple-label image classification. To download and explore this dataset, users are required to accept a Date Usage Agreement (DUA) called PhysioNet Credentialed Health Data License 1.5.0 (https://www.physionet.org/about/licenses/physionet-credentialed-health-data-license-150/). By accepting this DUA, users agree that the dataset can be used for scientific research and educational purposes only and will not attempt to re-identify any patients, institutions or hospitals. Additionally, the authors should cite this original paper for any publication that explores this dataset.

In this study, our objective is to provide an extensive open dataset of mammograms that include annotations from radiologists. We have used the consensus among radiologists as ground truth to ensure the reliability of the annotations. However, the VinDr-Mammo dataset has certain limitations such as the absence of pathology-confirmed ground truth data and other essential clinical information like molecular and histology data. As a result, it relies heavily on the expertise of radiologists. Biopsy tests are currently the most reliable means of measuring breast cancer. However, obtaining a significant number of mammographic images, each with a biopsy test, is impractical and outside the scope of this study. Given the data’s incomplete support from pathology reports, it should not be used to directly evaluate CAD for diagnosis purposes, but only used in training settings. For the screening purpose, the dataset can be directly used to evaluate CAD after converting the provided BI-RADS annotations in 5 categories to the 3-category system: BI-RAS 0 (recall, correspond to BI-RADS 3, BI-RADS 4, BI-RADS 5), BI-RAS 1 (normal, correspond to BI-RADS 1), BI-RAS 2 (benign, correspond to BI-RADS 2). Additionally, some abnormalities, such as skin and nipple retraction, have less than 40 samples, making studying these abnormalities on this dataset less reliable. Finally, the introduced dataset is not DICOM-compliant and it would fail to be processed properly by DICOM processing libraries.

## Supplementary information

Supplementary materials

## Data Availability

The codes used in this study were made publicly available. The scripts used for loading and processing DICOM images are based on the following open-source repositories: Python 3.8.0 (https://www.python.org/); Pydicom 1.2.0 (https://pydicom.github.io/); and Python hashlib (https://docs.python.org/3/library/hashlib.html). The code for data pseudonymization and stratification was made publicly available at https://github.com/vinbigdata-medical/vindr-mammo.
