# Supplementary material for: VinDr-Mammo: A large-scale benchmark dataset for computer-aided diagnosis in full-field digital mammography
Source: Sci Data. 2023 May 12;10:277. doi: 10.1038/s41597-023-02100-7 (PMC10182079; doi:10.1038/s41597-023-02100-7)
Supplement: Supplementary file 1 — Supplementary materials [file 41597_2023_2100_MOESM1_ESM.pdf]

Supplementary materials

Table 1. Definition of findings used in the study.

<table>
<tr><th colspan="2">Finding</th><th>Definition</th></tr>
<tr><td colspan="2">1. Mass</td><td>A mass is 3-dimensional and occupies space. It has completely or partially convex-outward borders and (when radiodense) appears denser in the center than at the periphery. If a potential mass is seen only on a single projection, it should be called an asymmetry until its 3-dimensionality is confirmed.</td></tr>
<tr><td colspan="2">2. Suspicious Calcification</td><td>Calcification with suspicious morphology (amorphous, coarse heterogeneous, fine pleiomorphic, fine linear, or fine linear branching) or probably benign (BI-RADS 3).</td></tr>
<tr><td rowspan="3">Asymmetries</td><td>3. Asymmetry</td><td>This is an area of fibroglandular-density tissue that is visible on only one mammographic projection. Most such findings represent summation artifacts, a superimposition of normal breast structures, whereas those confirmed to be real lesions (by a subsequent demonstration on at least one more projection) may represent one of the other types of asymmetry or a mass.</td></tr>
<tr><td>4. Global Asymmetry</td><td>Global asymmetry is judged relative to the corresponding area in the contralateral breast and represents a large amount of fibroglandular-density tissue over a substantial portion of the breast (at least one quadrant). There is no mass, distorted architecture, or associated suspicious calcifications.</td></tr>
<tr><td>5. Focal Asymmetry</td><td>A focal asymmetry is judged relative to the corresponding location in the contralateral breast, and represents a relatively small amount of fibroglandular-density tissue over a confined portion of the breast (less than one quadrant). It is visible on and has a similar shape on different mammographic projections (hence, a real finding rather than the superimposition of normal breast structures), but it lacks the convex-outward borders and the conspicuity of a mass. Rather, the borders of a focal asymmetry are concave-outward, and it is usually seen to be interspersed with fat.</td></tr>
<tr><td colspan="2">6. Architecture Distortion</td><td>The parenchyma is distorted with no definite mass visible. This includes thin straight lines or spiculations radiating from a point, and focal retraction, distortion, or straightening at the anterior or posterior edge of the parenchyma.</td></tr>
<tr><td colspan="2">7. Suspicious Lymph Node</td><td>Axillary lymph nodes receive lymph from vessels that drain the arm, the walls of the thorax, the breast, and the upper walls of the abdomen. Features of suspicious lymph nodes include loss or disruption of central fatty hilum, loss or pericapsular fat line, irregular outer margins, hyperattenuating, and calcified.</td></tr>
<tr><td colspan="2">10. Skin Thickening</td><td>Skin thickening may be focal or diffuse and is defined as being greater than 2 mm in thickness. This finding is of particular concern if it represents a change from previous mammography examinations. However, unilateral skin thickening is an expected finding after radiation therapy.</td></tr>
<tr><td colspan="2">11. Skin Retraction</td><td>The skin is pulled in abnormally.</td></tr>
<tr><td colspan="2">12. Nipple Retraction</td><td>The nipple is pulled in. This should not be confused with nipple inversion, which is often bilateral and which in the absence of any suspicious findings and when stable for a long period of time, is not a sign of malignancy. However, if nipple retraction is new, suspicion of underlying malignancy is increased.</td></tr>
</table>

Table 2. DICOM tags (a). The list of DICOM tags that were retained for loading and processing raw images. All other tags were removed for protecting patient privacy. Details about all these tags can be found from DICOM Standard Browser at https://dicom.innolitics.com/ciods.

DICOM Tag	Attribute Name	Description
(0010,0040)	Patient's Sex	Sex of named Patient.
(0010,1010)	Patient's Age	Age of the Patient.
(0010,1020)	Patient's Size	Length or size of the Patient, in meters.
(0010,1030)	Patient's Weight	Weight of the Patient, in kilograms
(0028,0010)	Rows	Number of rows in the image.
(0028,0011)	Columns	Number of columns in the image.
(0028,0030)	Pixel Spacing	Physical distance in the patient between the center of each pixel, specified by a numeric pair – adjacent row spacing (delimiter) adjacent column spacing, in mm.
(0018,1164)	Imager Pixel Spacing	Physical distance measured at the front plane of the Image Receptor housing between the center of each pixel. Specified by a numeric pair – adjacent row spacing (delimiter) adjacent column spacing, in mm.
(0028,0120)	Pixel Padding Value	Single pixel value or one limit (inclusive) of a range of pixel values used in an image to pad to rectangular format or to signal background that may be suppressed.
(0028,0121)	Pixel Padding Range Limit	Pixel value that represents one limit (inclusive) of a range of padding values used together with Pixel Padding Value (0028,0120) as defined above.
(0028,0100)	Bits Allocated	Number of bits allocated for each pixel sample. Each sample shall have the same number of bits allocated. Bits Allocated (0028,0100) shall be either 1, or a multiple of 8.
(0028,0101)	Bits Stored	Number of bits stored for each pixel sample. Each sample shall have the same number of bits stored.
(0028,0102)	High Bit	Most significant bit for pixel sample data. Each sample shall have the same high bit. High Bit (0028,0102) shall be one less than Bits Stored (0028,0101)
(0028,0103)	Pixel Representation	Data representation of the pixel samples. Each sample shall have the same pixel representation.
(2050,0020)	Presentation LUT Shape	Specified predefined Presentation LUT transformation. Required of Presentation LUT Sequence (2050,0010) is absent.
(0028,0106)	Smallest Image Pixel Value	The minimum actual pixel value encountered in this image.
(0028,0107)	Largest Image Pixel Value	The maximum actual pixel value encountered in this image.
(0028,1050)	Window Center	Window Center for display.
(0028,1051)	Window Width	Window Width for display.
(0028,1055)	Window Center & Width Explanation	Free form explanation of the meaning of the Window Center and Width. Multiple values corresponding to multiple Window Center and Width values.
(7FE0,0010)	Pixel Data	A data stream of the pixel samples that comprise the Image. Required if Pixel Data Provider URL (0028,7FE0) is not present.

Table 3. DICOM tags (b). The list of DICOM tags that were retained for loading and processing raw images. All other tags were removed to protect patient privacy. Details about all these tags can be found from DICOM Standard Browser at https://dicom.innolitics.com/ciods.

DICOM Tag	Attribute Name	Description
(0028,1056)	VOI LUT Function	Describe a VOI LUT function to apply to the values of Window Center (0028,1050) and Window Width (0028,1051).
(0028,3010)	VOI LUT Sequence	Defines a Sequence of VOI LUTs. One or more items shall be included in this Sequence. The required of Window Center (0028,1050) is not present. May be present otherwise.
(0028,3002)	LUT Descriptor	Specifies the format of the LUT Data in this Sequence.
(0028,3003)	LUT Explanation	Free form text explanation of the meaning of the LUT.
(0028,3006)	LUT Data	LUT Data in this sequence.
(0028,1052)	Rescale Intercept	The value b in the relationship between stored values (SV) and the output units. Output units = m*SV+b
(0028,1053)	Rescale Slope	m in the equation specified in Rescale Intercept (0028,1052).
(0028,1054)	Rescale Type	Specifies the output units of Rescale Slope (0028,1053) and Rescale Intercept(0028,1052).
(0028,0004)	Photometric Interpretation	Specifies the intended interpretation of the pixel data.
(0028,2110)	Lossy Image Compression	Specifies whether an Image has undergone lossy compression (at a point in its lifetime).
(0028,2112)	Lossy Image Compression Ratio	Describes the approximate lossy compression ratio(s) that have been applied to this image.
(0028,2114)	Lossy Image Compression Method	A label for the lossy compression method(s) that have been applied to this image.
(0028,0002)	Samples per Pixel	Number of samples (planes) in this image.
(0028,0008)	Number of Frames	Number of frames in a Multi-frame Image.
(0008,0018)	SOP Instance UID	Uniquely identifies the SOP Instance
(0020,000e)	Series Instance UID	Unique identifier of the Series containing the referenced Instances.
(0020,000d)	Study Instance UID	Unique identifier of the Study containing the referenced Instances.
(0008,0060)	Modality	Type of equipment that originally acquired the data used to create the images in this Series.
(0018,0015)	Body Part Examined	Text description of the part of the body examined.
(0008,0068)	Presentation Intent Type	Identifies the intent of the images that are contained within this Series
(0008,0070)	Manufacturer	Manufacturer of the device.
(0008,1090)	Manufacturer's Model Name	Manufacturer's model name of the device.
(0020,0060)	Laterality	Laterality of (paired) body part examined. Required if the body part examined is a paired structure and Image Laterality (0020,0062) or Frame Laterality (0020,9072) or Measurement Laterality (0024,0113) are not present.
(0020,0062)	Image Laterality	Laterality of (possibly paired) body part (as described in Anatomic Region Sequence (0008,2218)) examined.
(0018,0051)	View Position	Radiographic view of the image relative to the imaging subject's orientation.
